# All-Ceramic Fiber Fabry–Perot Cavity High-Temperature Pulsating Pressure Sensor Based on HTCC

**DOI:** 10.3390/s25123678

**Published:** 2025-06-12

**Authors:** Xiangcong Xu, Fei Wang, Guoqing Han, Huiyi Tang, Wanfeng Zhou, Xiaohua Lei, Xianming Liu

**Affiliations:** 1 Chongqing Materials Research Institute Co., Ltd., National Engineering Research Center for Instrument Functional Materials, Chongqing 400707, China; xiangcongxu@163.com (X.X.); hytang320@163.com (H.T.); 2 Key Laboratory of Optoelectronic Technology and Systems, Ministry of Education, Chongqing University, Chongqing 400044, China; xhlei@cqu.edu.cn (X.L.); xianming65@163.com (X.L.); 3 The 43rd Research Institute of China Electronics Technology Group Corporation, Hefei 235000, China; wang0o0fei@163.com (F.W.); zhouwanfeng126@126.com (W.Z.)

**Keywords:** pressure sensors, fast response, Fabry–Perot, all-ceramic, HTCC

## Abstract

In the aerospace, energy and nuclear energy sectors, dynamic pressure measurement of power equipment and pressure vessels in high-temperature environments is critical for validating design, manufacturing processes and operational condition monitoring. The existing electric sensors are resistant to temperature. It is difficult to meet the pressure measurement requirements of high temperature and high-frequency responses. In this paper, combining the material properties of high-temperature co-fired ceramics (HTCC) with the structural characteristics of Fabry–Perot, an all-ceramic fiber-optic Fabry–Perot high-temperature pulsating pressure sensor based on a HTCC pressure- sensing diaphragm and ceramic high-temperature sintering process, is proposed. Experimental results show that in the pressure range of 6 MPa, the static pressure sensitivity of the sensor is 1.30 nm/MPa, and the linear goodness of fit reaches 0.99913. The dynamic response frequency of the sensor reaches 598.5 kHz. The survival time at high temperature of 800 °C is more than 80 h. The sensitivity to temperature is 0.00475 nm/°C.

## 1. Introduction

In the fields of aerospace, energy industry and nuclear energy, high-speed dynamic pressure will be generated in key power equipment, such as aero-engines [1], which are often accompanied by high temperature and strong electromagnetic interference. Common electrical pressure sensors based on the piezoelectric effect, piezoresistive effect and capacitance principle have shortcomings in temperature resistance [2]. The optical fiber sensor has the advantages of simple principle, small volume, high sensitivity, anti-electromagnetic interference and resistance to extreme environment [3]. Therefore, in recent years, researchers have introduced optical fiber sensors into the measurement of parameters, such as temperature [4], pressure [5], strain [6], and clearance [7], in similar extreme environments.

The field of pressure sensing mainly uses sensors based on the principle of optical fiber Fabry–Perot interference. According to the difference of the Fabry–Perot cavity medium, the common optical fiber Fabry–Perot pressure sensor can be divided into intrinsic and extrinsic [8]. For the intrinsic optical fiber Fabry–Perot pressure sensor, Wang et al. fabricated an intrinsic fiber Fabry–Perot pressure sensor composed of a gold–parylene–gold three-layer material at the fiber end using vacuum deposition technology for underwater shock wave pressure testing. The rise time of an underwater shock wave is less than 0.767 μs. It can withstand more than eight tests of 30 MPa pressure [9]. The same sensor manufactured by Cranch et al. enables the measurement of shock dynamic pressure of 60 MPa in actual shock wave tests [10]. However, the sensor of this structure uses flexible material, such as the Fabry–Perot cavity. A common problem of flexible materials is that they are not resistant to high temperature, and the tolerance temperature is generally not more than 400 °C [11]. For the extrinsic optical fiber Fabry–Perot pressure sensor, Sweeney et al. developed a metal diaphragm optical fiber Fabry–Perot pressure sensor. The sensor is used for dynamic pressure measurement in nuclear reactors or other harsh environments. It can realize the dynamic pressure measurement of ±270 kPa with the developed demodulation system [12]. At the same time, He et al. made an all-fiber microcavity pressure sensor for cylinder pressure measurement of internal combustion engines. The sensor can stably measure pressure in the temperature range of 40 °C to 200 °C. The resonant frequency of the sensor exceeds 10 kHz [13]. However, the above structures still have problems, such as small measurable pressure range, poor temperature resistance and low frequency response.

In order to improve the performance parameters of the sensor, such as temperature resistance, frequency response and voltage resistance, the current research mainly focuses on the improvement of sensor materials and the integrated process [14]. Among them, the metal material diaphragms represented by various types of stainless steel have good mechanical properties and relatively simple processing technology. However, under different temperature gradients, the mechanical properties of the metal change greatly, and the thermal expansion coefficient of the material is high. It has a greater temperature sensitivity under high-temperature conditions [15]. Therefore, the metal diaphragm sensor is difficult to meet the needs of measurement fields with high accuracy requirements. In addition, the metal film is inevitably oxidized at high temperatures, resulting in a decrease in surface reflectance. This causes the spectral contrast and intensity to be seriously reduced, and at the same time produces spectral distortion [16]. Crystal material diaphragms, such as monocrystalline silicon and sapphire, have good temperature resistance and mechanical properties. Crystal material diaphragms, such as monocrystalline silicon and sapphire, have been widely used in the field of sensors due to their good temperature resistance and mechanical properties [17]. However, diaphragm fabrication and sensor integration based on these materials often require complex processes, such as MEMS, high-temperature pressure bonding, and special laser welding [18]. Although the sensor has the advantages of high precision, the overall process is complex and the manufacturing process takes a long time. At the same time, the yield and output of a single production are limited, resulting in high manufacturing costs [19]. Diamond thin-film materials have the advantages of high hardness, low thermal expansion coefficient and ultra-high thermal conductivity. This makes it perform well in high temperatures and harsh environments [20]. However, the preparation process is complicated and the processing is difficult. The surface of the diamond is prone to carbonization in high-temperature environments, and stability in high-temperature environments cannot be effectively guaranteed. As a third-generation semiconductor material, silicon carbide material has excellent high-temperature stability and chemical stability [21]. At the same time, it has good radiation resistance [22]. In particular, sintered silicon carbide fabricated by laser processing technology has great potential in the field of high-temperature pressure sensing [23]. However, its preparation process is complex and the cost is high, and its packaging is not reliable under high-temperature, high-pressure and strong-vibration conditions.

High-temperature co-fired ceramics (HTCC) is a new type of ceramic material, which is suitable for the integrated packaging of high-power microelectronic devices with high- thermal stability and mechanical-strength requirements [24]. At present, there have been reports on the application of HTCC to the development of pressure sensors. It is mainly based on its thermodynamic properties and the characteristics of the HTCC process itself that can realize the printing of electrodes on the surface or in the stack [25]. For example, the HTCC capacitive pressure sensor is made, and its core working principle is that the substrate printed with electrodes will undergo deflection deformation under pressure, change the capacitance value in the chamber, and realize pressure sensing [26]. Compared with the same structure sensor using other materials, its temperature resistance performance is much better. The above research shows that HTCC materials have the potential to resist high temperature and high pressure in the sensor field, and can meet the stable working requirements in extreme environments.

In this paper, the HTCC diaphragm is applied to the fabrication of the diaphragm fiber Fabry–Perot pressure sensor for the first time. The whole ceramic pressure-sensing element structure is adopted. A scheme of all-ceramic fiber Fabry–Perot pressure sensor based on HTCC for dynamic pressure measurement in high-temperature environments is proposed. It is applied to dynamic pressure measurement in high-temperature environments. Some research studies on sensor structure design have been carried out in the early stages [27]. Based on the HTCC process, the ceramic substrate, ceramic insert and gold-plated optical fiber were sintered into an integrated ceramic Fabry–Perot cavity pressure-sensing element by the high-temperature sintering process. At the same time, the probe-type metal packaging was carried out. The sensor can withstand pressures up to 6 MPa, with a dynamic response frequency reaching 598.5 kHz, and is capable of long-term operation at 800 °C. The experimental results show that the all-ceramic fiber Fabry–Perot pressure sensor based on HTCC proposed in this paper has the characteristics of a simple structure, low cost and easy preparation. It provides a feasible scheme for dynamic pressure measurement in many harsh environments.

## 2. Sensor Principle and Design

### 2.1. Structural Design

**Figure 1:**
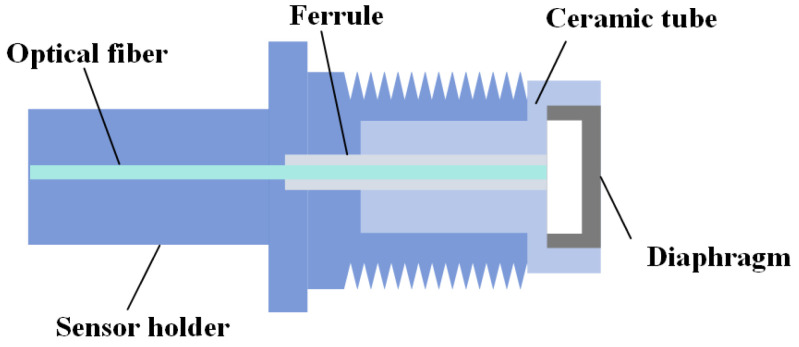
Structure diagram of a HTCC optical fiber Fabry–Perot pressure sensor.

The structure of an optical fiber Fabry–Perot pressure sensor is relatively simple, and it is generally composed of a sensor bracket, optical fiber core, ceramic tube, single mode fiber and HTCC pressure-sensing diaphragm. The specific structure diagram is shown in Figure 1:

A Fabry–Perot cavity is formed between the pressure-sensing diaphragm with microstructure and the upper surface of the plug. When the pressure is applied, the diaphragm in the free state produces deflection deformation to realize the perception of pressure. The single-mode fiber is aligned to the diaphragm through the insert, and the fiber end face and the inner surface of the diaphragm form two reflective surfaces of the fiber Fabry–Perot structure. After the optical element is assembled, it is encapsulated by a metal bracket to provide protection and support for the sensor.

The common single-mode fiber coating material is acrylate, and its temperature resistance usually does not exceed 150 °C. Although the quartz optical fiber itself can withstand higher temperatures, the bare optical fiber without coating protection will break due to thermal strain during heating or cooling. It will make the sensor fail. In order to improve the temperature resistance of the sensor, gold-plated fiber is used instead of quartz single-mode fiber in the design of this paper. The coating layer on the surface of the gold-plated fiber is gold, which has higher temperature resistance, and its bending resistance and tensile resistance are also significantly improved. The pressure sensitive diaphragm material of the sensor designed in this paper is HTCC. In order to avoid the large error caused by the different thermal expansion coefficients of each component to the sensor in the high-temperature pressure measurement environment, the optical fiber plug is also selected as the ceramic material. Combined with the size characteristics of the gold-plated optical fiber and pressure sensitive diaphragm, we customized a ceramic insert with a purity of 99% alumina as a collimating device for optical fiber.

**Figure 2:**
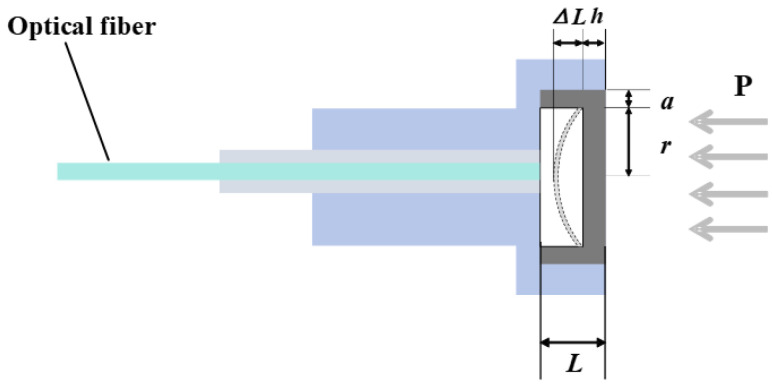
Diaphragm optical fiber pressure sensor pressure-sensing principle diagram.

The sensitive diaphragm plays a key role in the sensor and has an impact on the performance of the sensor. Therefore, the key to the design of the sensor is to design the pressure-sensing diaphragm of the sensor. As shown in Figure 2, the schematic diagram of the sensing diaphragm deflection deformation of the diaphragm type-fiber optic Fabry–Perot pressure sensor is demonstrated. It is used to illustrate the mechanical modeling of the change in cavity length of a circular thin-film sheet under pressure.

In the figure, r is the effective sensing radius of the circular diaphragm. h is the thickness of the diaphragm. *P* is the pressure loaded on the diaphragm by the outside world. Δ*L* is the deflection change in the center of the diaphragm under pressure, which is also expressed as the change in the cavity length of the sensor. The specific expression is [28]:

(1) $$\Delta L=\frac{3r^{4}(1-\nu^{2})}{16Eh^{3}}\cdot P,$$

In Equation (1), *E* is the elastic modulus of the diaphragm material, *ν* is the Poisson’s ratio of the material, and *ρ* is the density of the material.

The natural frequency of the diaphragm is one of the important parameters of the dynamic pressure sensor, which determines the dynamic response characteristics of the sensor. For the circular thin film, the natural frequency expression is [29]:

(2) $$f=\frac{a_{mn}}{4\pi }\cdot \frac{h}{r^{2}}\sqrt{\frac{E}{3\rho (1-\nu^{2})}},$$

In Equation (2), $a_{mn}$ is the frequency coefficient of the clamped circular thin plate, and its size is independent of the material parameters. In the natural frequency of the sensor, the first-order frequency is mainly concerned, and the value of the first-order frequency coefficient $a_{mn}$ is approximately 10.215. It can be seen from the above formula that the natural frequency of the diaphragm is its own physical characteristics. It is only related to the structure, fastening method and material properties of the diaphragm, and has nothing to do with the external load applied to the diaphragm. Therefore, the key to affecting the natural frequency of the diaphragm is the design of the diaphragm size.

The key dimensions of the diaphragm are the effective pressure-sensing radius *r* and the diaphragm thickness *h*. According to the theory of small deflection deformation, the maximum deflection deformation of the center of the pressure-sensitive diaphragm under pressure should be less than 1/5 of the diaphragm thickness *h*.

(3) $$\Delta L_{\max}=\frac{3r^{4}(1-\nu^{2})}{16Eh^{3}}\cdot P<\frac{h}{5},$$

At the same time, the material for making the diaphragm has a mechanical performance limit. When the deflection deformation of the diaphragm exceeds a certain value, the diaphragm will be damaged due to stress concentration. It should be ensured that the stress on the surface of the diaphragm should be less than the failure stress of the material when the diaphragm is subjected to the maximum pressure of the range [30]. Generally, this stress is less than 0.8 times the failure stress in design.

(4) $$\sigma_{\max}=\frac{3r^{2}}{14h^{2}}\cdot P<0.8\sigma_{bs},$$

In Equation (4), $\sigma_{\max}$ is the maximum surface stress at the maximum deflection deformation of the diaphragm, and $\sigma_{bs}$ is the failure stress of the diaphragm material.

We substitute the maximum withstand pressure *P* of the sensor of 6 MPa into the above limiting conditions for calculation. The range of the ratio of the thickness *h* to the effective radius *r* in the design of the diaphragm is obtained.

(5) $$0.07\leq \frac{h}{r}\leq 0.4,$$

The external packaging structure of the fiber optic pressure sensor is the standard M5 thread size, and the diameter is not more than 5 mm. The diameter of the M5 coarse thread is 4.3 mm. In order to ensure the smooth installation of the sensor, the diameter of the sensor end should not be greater than this size. In case of the need to reserve the wall thickness of the external packaging structure bracket, the overall outer diameter of the diaphragm should not be greater than 2.5 mm. In order to balance the bonding strength and processing difficulty, the effective radius of the diaphragm is set to 0.75~0.1 mm. According to the lamination process of the HTCC diaphragm, the thickness range is set to 75~100 μm.

**Figure 3:**
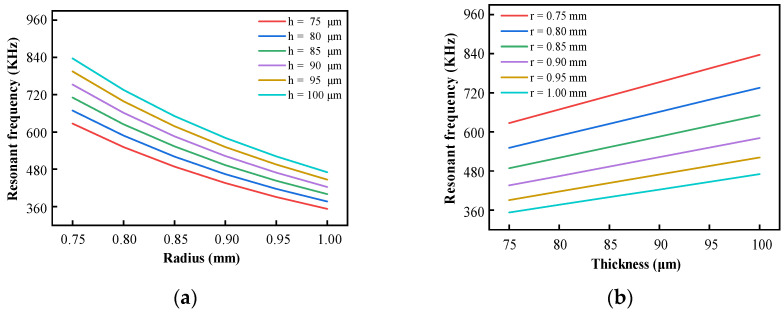
Relationship between the *f* of the diaphragm and *r* or *h*: (**a**) Relationship between frequency and effective radius; (**b**) Relationship between frequency and effective thickness.

According to Equation (2), the natural frequency of the corresponding size is obtained. The comparison results are shown in Figure 3.

According to the results shown in Figure 3, the natural frequency of the diaphragm decreases with the increase in the effective radius *r* and increases with the increase in the effective thickness *h*. Within the allowable size range, the natural frequencies of the diaphragm meet the design requirements. Therefore, in the design of the diaphragm, the value of the effective radius *r* can be increased within a certain range, and the value of the effective thickness *h* can be reduced to improve the sensitivity of the sensor as much as possible under the premise of meeting the minimum frequency response index.

Sensitivity is usually defined as the ratio of the sensor output value to the input value. For the diaphragm optical fiber Fabry–Perot pressure sensor, the sensitivity *S* can be expressed as:

(6) $$S=\frac{\Delta L}{\Delta P}=\frac{3r^{4}(1-\nu^{2})}{16Eh^{3}},$$

**Figure 4:**
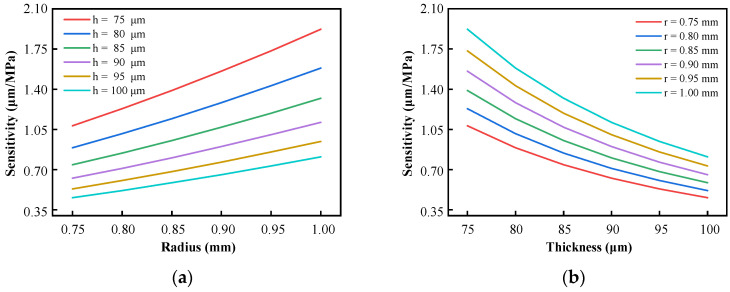
Relationship between the *S* of the diaphragm and *r* or *h*: (**a**) Relationship between sensitivity and effective radius; (**b**) Relationship between sensitivity and effective thickness.

According to Equation (6), the numerical simulation results of the relationship between the sensitivity *S* of the sensor and the effective radius *r* and the effective thickness *h* of different diaphragms are shown in Figure 4.

It can be observed from Figure 4 that when the effective radius *r* of the diaphragm is larger and the effective thickness *h* is smaller, the theoretical sensitivity of the sensor is higher. In particular, HTCC uses the laminated process of raw ceramic sheets to achieve the thickness fabrication of the designed structure. The thickness of the raw porcelain is usually fixed. According to different process characteristics, the common thickness of HTCC green porcelain is 25 μm, 50 μm, and 100 μm. Other thicknesses require laminated thin raw ceramic tape, interlayer alignment and interface bonding are more difficult, and the process complexity increases. The 100 μm corresponds to a single-layer standard green tape, and the process is simpler and more reliable. Therefore, the effective thickness *h* is set to 100 μm. Considering the support strength, the diaphragm width is not less than 0.3 mm, take 0.35 mm. When the outer diameter of the diaphragm is 2.5 mm, the effective pressure-sensing radius of the diaphragm is 0.90 mm.

The curvature of the Fabry–Perot cavity will lead to a decrease in the spectral quality factor. In order to determine whether the decrease in the spectral quality factor caused by the compression of the diaphragm is significant, it is necessary to theoretically calculate the curvature radius of the diaphragm when the pressure-sensitive diaphragm is subjected to pressure load.

Under the above size conditions, combined with Equation (1), the theoretical maximum cavity length change in the diaphragm under 6 MPa pressure load is calculated to be 3.17 μm, as shown in Equation (7):

(7) $$\Delta L_{6\mathrm{MPa}}=\frac{3r^{4}(1-\nu^{2})}{16Eh^{3}}\cdot P=3.17\;\mu m,$$

For the circular diaphragm with fixed-edge support, the radius of curvature *R* of the diaphragm satisfies Equation (8) in the case of small deflection. The curvature radius of the diaphragm is calculated to be 127.7 mm. The effective pressure-sensing radius of the diaphragm is much smaller than the curvature radius. It shows that the diaphragm can be approximated as a plane at this time.

(8) $$R^{2}={\left(R-\Delta L\right)}^{2}+h^{2},$$

**Figure 5:**
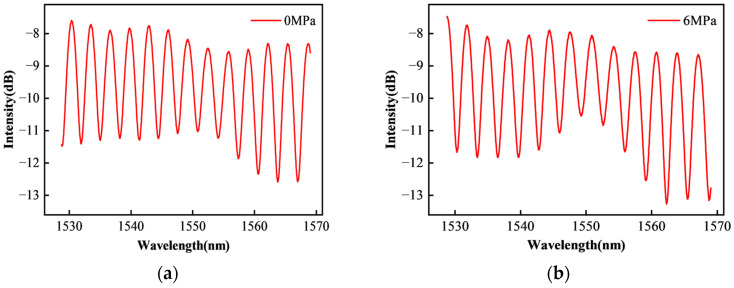
Sensor spectral signal: (**a**) initial state; (**b**) 6 MPa pressure state.

At the same time, the single-mode fiber was used in the system designed in this paper. Compared with multimode fiber, the light intensity is more concentrated and the fluctuation is smaller. Therefore, the influence of local changes caused by ∆*L* on the overall spectral signal quality can be approximately ignored. In order to confirm this view more clearly, this paper compares the spectral signals of the sensor in the initial state and the maximum pressure of 6 MPa, as shown in Figure 5. It can be seen that the spectrum is only shifted on the wavelength before and after the compression, and the spectral signal strength is basically unchanged.

Therefore, the effective pressure-sensing radius, diaphragm thickness and other parameters are comprehensively considered. In this paper, the specific size of the pressure-sensitive diaphragm is designed. It is confirmed that the influence of the spectral signal quality after the pressure change in the diaphragm can be ignored, thereby ensuring that the diaphragm can improve the range, sensitivity and resonance frequency of the sensor under the premise of ensuring the small size as far as possible.

### 2.2. Sensor Characteristic Analysis

The frequency response characteristic is an important parameter of the optical fiber pressure sensor studied in this paper. After completing the design of the HTCC diaphragm, the frequency analysis function in SolidWorks Simulation (SolidWorks 2022 SP5.0) is used to analyze it theoretically. We can obtain the resonant response characteristics of the diaphragm at the design size. This provides a theoretical basis and an intuitive dynamic change process for carrying out dynamic experiments to evaluate the actual frequency response performance of the sensor. At the same time, the pulsating pressure sensor should be applied to high- temperature environments, which requires the sensor to have excellent and stable temperature resistance. In a high-temperature environment, the thermal expansion effect of the material and the mechanical properties of each component of the sensor will change with temperature. It will lead to a deviation between the performance of the sensor in a high-temperature environment and that at room temperature; thus, affecting the accuracy and sensitivity of the sensor. Therefore, it is also very important to analyze the characteristics of the sensor in a high-temperature environment.

#### 2.2.1. Transient Response Analysis

In the dynamic performance experiment of the sensor, the shock tube and other equipment are mainly used to apply transient impact pressure to the sensor [31,32].

The sensor designed in this paper must ensure high sensitivity while withstanding 6 MPa pressure and maintaining a 300 kHz resonant frequency. Combined with Section 2.1, the corresponding relationship between the natural frequency and sensitivity of the diaphragm and the diaphragm thickness *h* and the effective pressure-sensing radius *r*, is discussed. We selected reasonable numerical simulation parameters. Therefore, the transient pressure response analysis of the diaphragm is carried out for the sensor in this paper.

**Figure 6:**
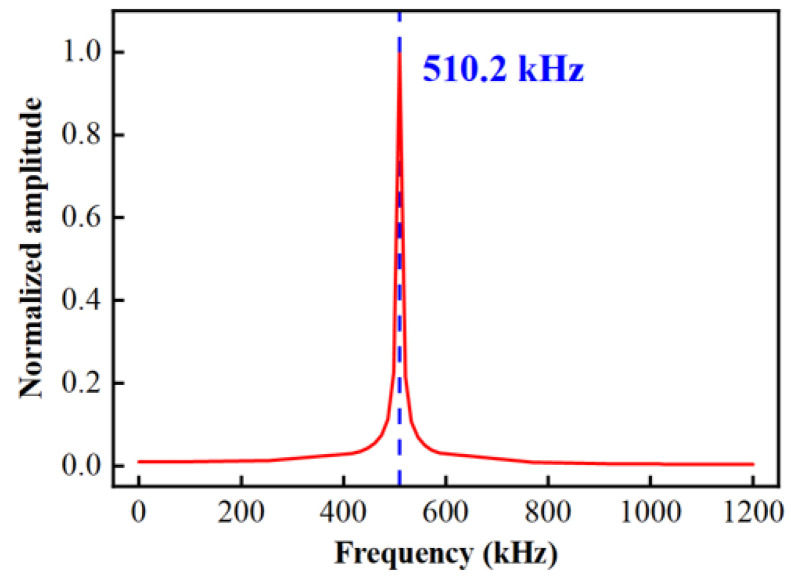
Harmonic response analysis results of HTCC diaphragm.

In this paper, the transient response finite element simulation method is used to explore the surface oscillation of the diaphragm when it is subjected to transient impact. For simulation, a shock transient pressure excitation is constructed and applied to the diaphragm surface. The response curve of the surface vibration amplitude is analyzed when the diaphragm resonates under the excitation. According to the theory of mechanical vibration, steady-state resonance can only be excited when the excitation frequency is greater than or equal to the first-order natural frequency of the system. The harmonic response analysis method is used for simulation calculation. The external load applied on the surface of the diaphragm is set as a dynamic load. The applied frequency increases from 1 Hz to the set upper limit of frequency scanning over time. The harmonic response analysis results of the HTCC diaphragm are shown in Figure 6.

**Figure 7:**
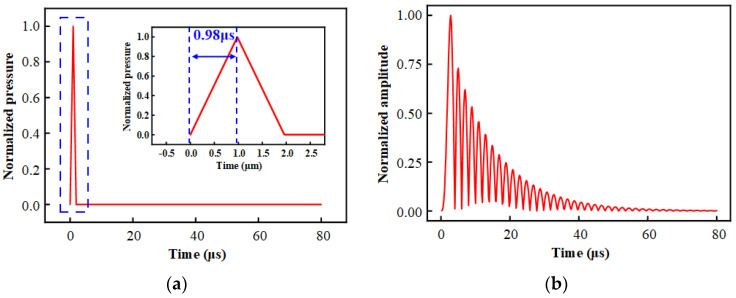
Simulation results of the response of HTCC diaphragm to transient pressure: (**a**) Constructed transient pressure excitation; (**b**) Resonance response curve of the surface amplitude of the diaphragm under transient pressure excitation.

According to Figure 6, the resonant frequency of the diaphragm designed in this paper is 510.2 kHz. Therefore, the critical value corresponding to the first-order resonant period is 1.96 μs. The rise time (t_r_) of the pulse signal needs to be less than 1/2 period to fully excite the modal response. Therefore, the rise time and fall time of the excitation are set to be 0.98 μs, which satisfies the critical condition. In this way, the signal distortion is avoided and the energy effective input is ensured. The excitation signal and simulation results are shown in Figure 7.

From the simulation results, it can be seen that the excitation acting on the surface of the diaphragm produces a resonant wave with a pronounced period. At the same time, the resonance phenomenon gradually decays and disappears with time. This is because we consider the damping effect when the sensor is actually working in the simulation, and we add damping to the simulation model. The time domain response curve of the diaphragm surface amplitude shows obvious characteristics of the underdamped system.

At the same time, the resonant frequency of the diaphragm under the design size is calculated by using Equation (2). The theoretical resonant frequency of the diaphragm is 580.9 kHz, as shown in Equation (9):

(9) $$f=\frac{a_{mn}}{4\pi }\cdot \frac{h}{r^{2}}\sqrt{\frac{E}{3\rho (1-\nu^{2})}}=580.9\;kHz,$$

By comparing the finite element simulation and theoretical calculation of the diaphragm designed in this paper, it is found that there are some differences between the two results. However, the values obtained by the two methods are much larger than the 300 kHz required by the sensor design in this paper. The designed diaphragm fully meets the requirements.

#### 2.2.2. High-Temperature Performance Analysis

In view of the high-temperature deformation characteristics of the whole sensor, the coupling and heat transfer characteristics between the components need to be considered. This paper focuses on the analysis of the deformation characteristics of the designed HTCC pressure sensitive diaphragm in a high-temperature environment using finite element software to apply a combination of load and temperature to the diaphragm to calculate the deflection deformation of the diaphragm.

Considering the packaging structure of the sensor designed in this paper, the edge of the HTCC pressure-sensitive diaphragm is rigidly fixed. Only the pressure surface is left exposed to the outside. This determines that the edge of the diaphragm is completely fixed during the diaphragm simulation. At this time, under the action of thermal load, the diaphragm with fixed boundary is expanded by heat, and the diaphragm bulges to the outside of the Fabry–Perot cavity. Under the action of the pressure load, the external pressure directly acts on the boundary-constrained diaphragm, resulting in its depression to the inner side of the Fabry–Perot cavity.

**Figure 8:**
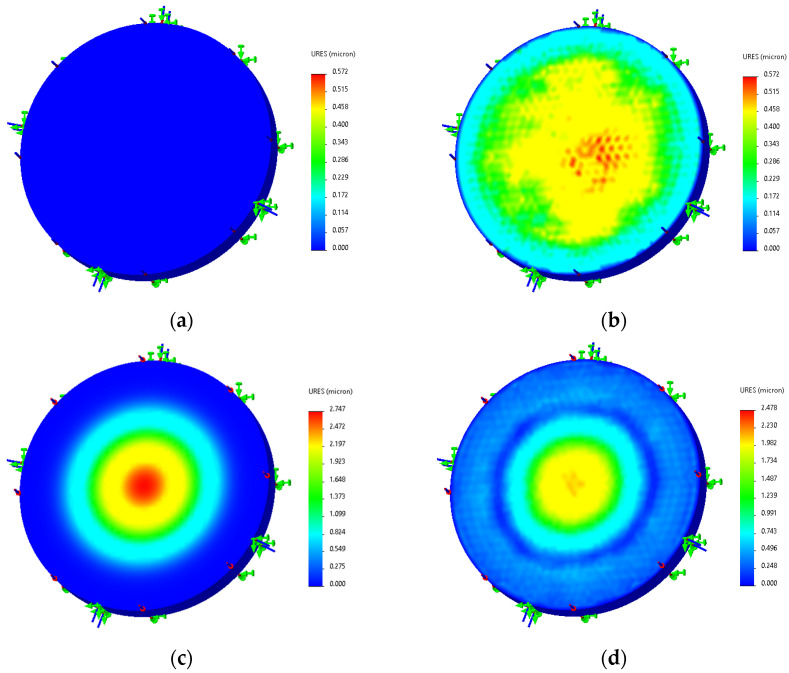
Deformation of HTCC diaphragm under free state and 4 MPa pressure: (**a**) Free state at 25 °C; (**b**) Free state at 500 °C; (**c**) 4 MPa load at 25 °C; (**d**) 4 MPa load at 500 °C.

Figure 8 shows the deflection of the diaphragm at room temperature (25 °C) and high temperature (500 °C) under free state (i.e., without pressure loading) and uniform load of 4 MPa.

From the simulation results, the diaphragm in the free state produces a thermal expansion effect in the high-temperature environment of 500 °C. The center of the diaphragm produces a deflection deformation of about 0.572 μm compared to room temperature. When the surface is loaded with 4 MPa pressure, the center of the diaphragm produces a deflection deformation of about 2.747 μm compared with the free state at room temperature. Analyzing the simulation results, it can be seen that the diaphragm in the free state produces a positive deflection change with respect to the bottom of the diaphragm in the temperature field, while the pressure effect produces a negative deflection change. Therefore, under the combined action of force and heat, the positive deflection caused by temperature will partially offset the negative deflection caused by pressure, resulting in a decrease in sensor sensitivity.

**Figure 9:**
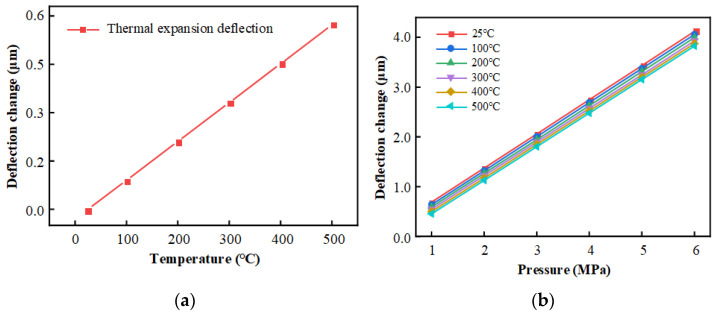
Changes in the center deflection of HTCC diaphragm under the combined action of force and heat: (**a**) Free state; (**b**) Combined action of force and heat.

Figure 9 demonstrates the variation in HTCC diaphragm center deflection under different conditions. According to the results in Figure 9a, the sensitivity of the diaphragm in the free state to the variation in its center deflection with temperature is 0.11550 nm/°C. According to the results in Figure 9b, the variation curve of diaphragm center deflection with pressure under the force-thermal compounding effect gradually shifts downward with the increase in temperature. It can be seen that in a high-temperature environment, the sensor sensitivity decreases compared to room temperature. Specifically, in the high-temperature environment of 500 °C, the pressure sensitivity caused by temperature is reduced by about 0.01155 μm/MPa, which is reduced by about 1.682%. It can be seen that the influence on the performance of the sensor is not significant in the high-temperature environment.

Accordingly, the sensor corresponding to the pressure-sensing diaphragm of this structure can produce a change in cavity length of 3.722 μm at 0–6 MPa. The theoretical resonance frequency is greater than 500 kHz. High-temperature environments do not have a significant effect on sensor performance.

## 3. Processing and Packaging

### 3.1. Pressure-Sensitive Diaphragm Preparation

**Figure 10:**
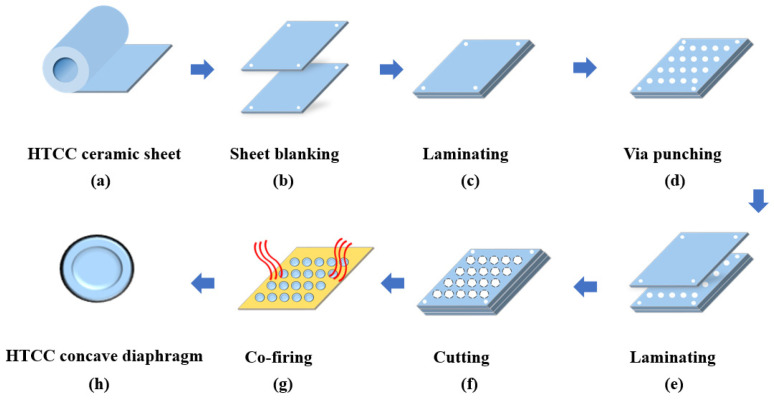
Processing flow of HTCC concave pressure-sensing diaphragm.

HTCC is a mature process [33], and its conventional process is mainly aimed at planar circuit substrates. The diaphragm designed in this paper is different from the conventional flat diaphragm, and the structure is a concave circular diaphragm. In the preparation process, the corresponding lamination, cutting and other processes need to be added. Moreover, the diaphragm size is small, and the machining process accuracy is higher. Combined with the structure and parameter characteristics designed in this paper, the processing flow of HTCC concave pressure sensitive diaphragm is shown in Figure 10.

**Figure 11:**
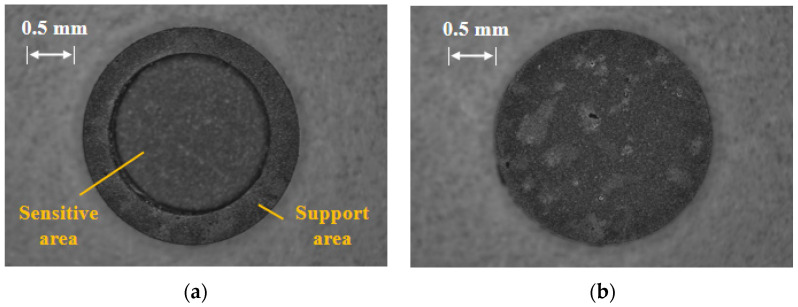
Picture of the processed HTCC diaphragm: (**a**) Enlarged view of front side; (**b**) Enlarged view of back side.

Finally, the HTCC pressure-sensitive diaphragm is made as shown in Figure 11.

### 3.2. Optical Performance Test and Optimization of Pressure-Sensitive Diaphragm

The HTCC diaphragm is not only used to sense pressure in the sensor, but also acts as an interference reflector. The quality of the interference spectrum directly affects the key performance parameters of the sensor, such as test accuracy, linearity and repeatability. The surface reflectivity of the diaphragm is the main factor affecting the spectral quality, which determines whether the HTCC diaphragm is suitable for manufacturing optical sensors.

**Figure 12:**
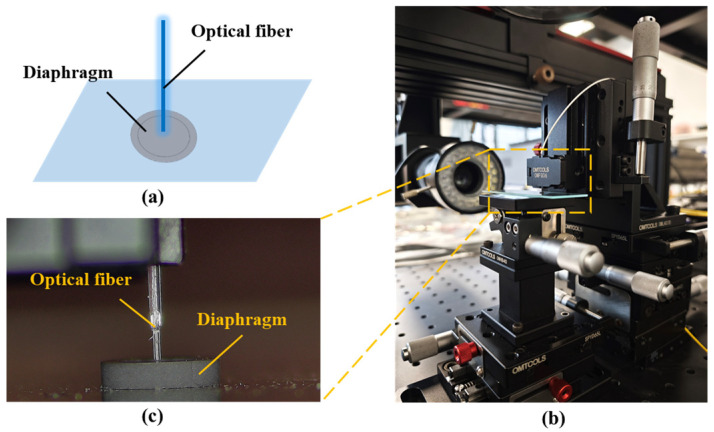
HTCC surface reflection characteristics test: (**a**) Test principle; (**b**) Actual test system; (**c**) Enlarged view of coupling area.

In order to study the surface reflectivity of the diaphragm sample, a simple test system was built in this paper. We used a single-mode quartz fiber with a flat end face to form a Fabry–Perot cavity with the inner surface of the diaphragm sample, and used a spectrometer to collect the reflected interference signal. We evaluated the quality of the reflection spectrum to determine the surface reflectivity of the sample. The test system and the object are shown in Figure 12.

**Figure 13:**
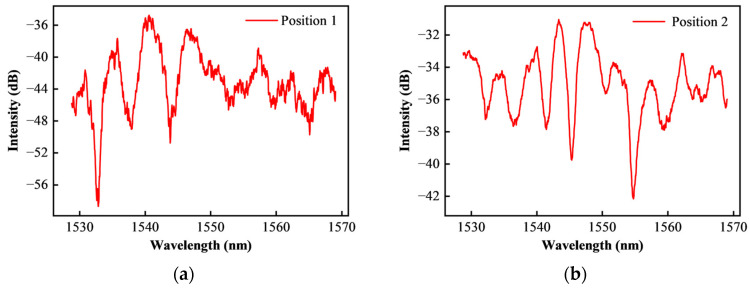
Experimental results of initial reflection characteristics of HTCC diaphragm: (**a**) Measuring position 1; (**b**) Measuring position 2.

Using the above method, the initial spectral quality test results of HTCC diaphragm are shown in Figure 13.

It can be seen from Figure 13 that the interference spectrum reflected from the different points on the surface of the diaphragm shows uneven spectrum, low intensity and serious burr phenomenon. The test results show that the untreated HTCC diaphragm is almost unusable in the fabrication of optical fiber sensors. This is because the initial surface roughness of the diaphragm is high, resulting in serious diffuse reflection of incident light on the surface. It affects the spectral signal formed by the reflection of the fiber end face.

**Figure 14:**
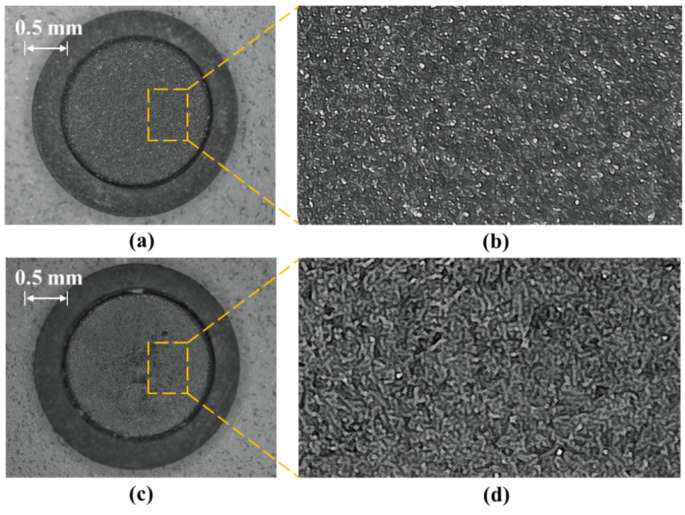
Surface characterization of HTCC diaphragm before and after polishing: (**a**) Before polishing; (**b**) Partial picture before polishing; (**c**) After polishing; (**d**) Partial picture after polishing.

In this paper, the inner surface of HTCC pressure sensitive diaphragm is treated by grinding and polishing. The surface reflection characteristics of the HTCC diaphragm are optimized by reducing the surface roughness of the reflector. Figure 14 shows the surface feature micrograph of the diaphragm before and after grinding and polishing.

According to Figure 14, it can be clearly seen that the surface characteristics are significantly improved after grinding and polishing. The roughness and graininess of the film surface decreased significantly. It shows smooth, uniform and flat appearance characteristics.

**Figure 15:**
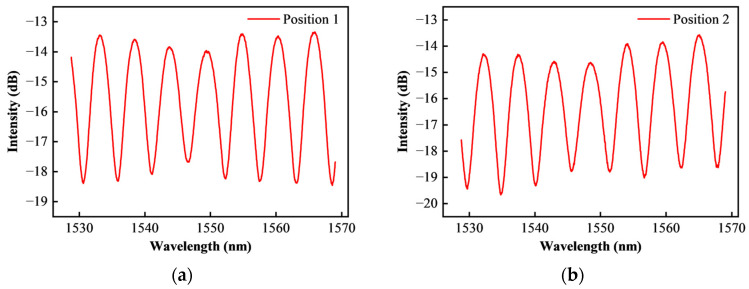
Experimental results of HTCC diaphragm surface grinding reflection characteristics: (**a**) Measuring position 1; (**b**) Measuring position 2.

After grinding and polishing, the interference spectrum test results formed by the diaphragm reflection surface are shown in Figure 15.

It can be seen from Figure 15 that the quality of the reflection spectrum of the HTCC pressure-sensitive diaphragm has been significantly improved after surface grinding. The uniformity, intensity and signal-to-noise ratio of the spectrum have been significantly improved. This greatly improves the demodulation accuracy and availability of the sensor.

### 3.3. Integration and Packaging

**Figure 16:**
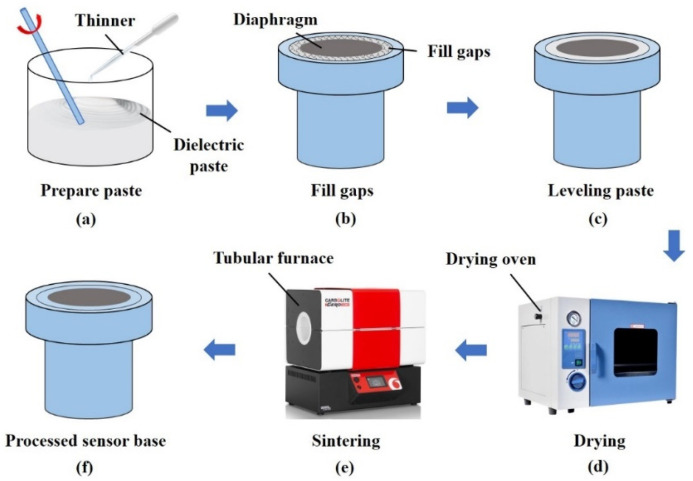
Integrated sintering process flow chart of the pressure-sensing element.

Since the pressure-sensing diaphragm is HTCC ceramic, in order to realize the all-homogeneous material of the Fabry–Perot pressure-sensing structure, combined with the ceramic process and the needs of high-temperature applications, an all-ceramic pressure-sensing element integration method based on the high-temperature sintering process is proposed, including the design and processing of ceramic tubes made of 99% alumina material as a substrate for carrying and sintering fixed HTCC inductive diaphragms. The ceramic tube consists of two stages of cylinders. The first stage of the cylinder has an outer diameter of 4.20 mm and a groove with a diameter of 3.20 mm and a depth of 0.40 mm is machined in the center to hold the diaphragm and to fill it with ceramic paste for sintering. The second-stage cylinder is used for inlay assembly with sensor housing and has an outside diameter of 3.00 mm. A 1.30 mm diameter through-hole is machined in the center of the ceramic tube for mounting the ceramic fiber optic ferrule. The sintering process flow of the ceramic tube and the inductive diaphragm is shown in Figure 16.

**Figure 17:**
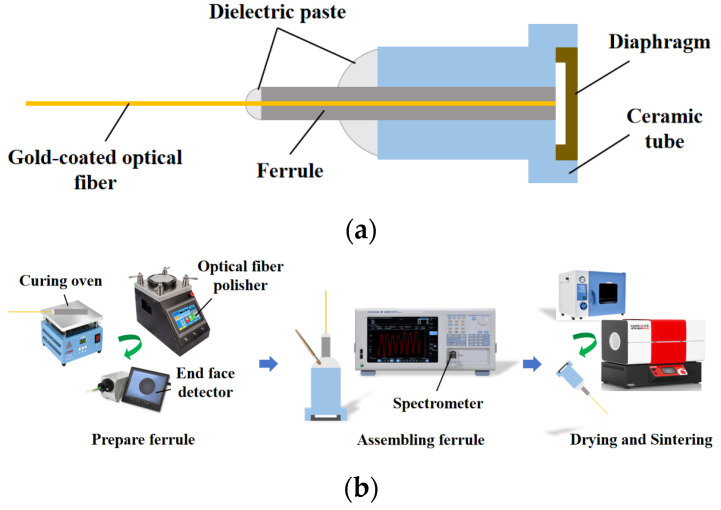
Full ceramic F–P pressure-sensing element structure and integration process: (**a**) Full ceramic pressure-sensing element structure; (**b**) Pressure-sensing element integration process flow.

The dielectric paste is used as the main raw material. Dielectric paste is a kind of electronic functional material made of glass powder and metal oxide powder, which is mainly used for thick film circuit multilayer wiring isolation and electronic component packaging [34]. The scientific high-temperature sintering process forms dense, high-temperature resistance, high hardness and strong bonding strength of the glass phase or ceramic phase of the dielectric layer. It has the effects of isolation and connection [35]. The same method is used to sinter the ferrule and the gold-coated optical fiber with the end face, as shown in Figure 17.

**Figure 18:**
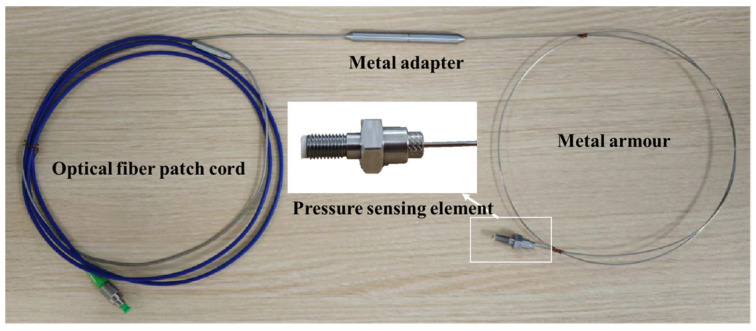
Metal packaging of the all-ceramic fiber Fabry–Perot pressure sensor based on HTCC.

One-piece sintering causes air to be retained inside the sensing element chamber, which expands and exits at high temperatures. This can lead to air gaps in the sintered area between the diaphragm and the ceramic tube, which in turn affects the airtightness of the resulting sensor. Therefore, the experimental process of sintering the HTCC diaphragm and the ceramic tube to form the substrate, and then sintering the fiber optic insert and the inductive element substrate, was adopted. The interface of the pressure sensor is the M5 thread. The sintered all-ceramic fiber optic Fabry–Perot cavity structure, as well as the metal-encapsulated pressure sensor, are shown in Figure 18.

## 4. Results and Discussion

### 4.1. Static Experiment

**Figure 19:**
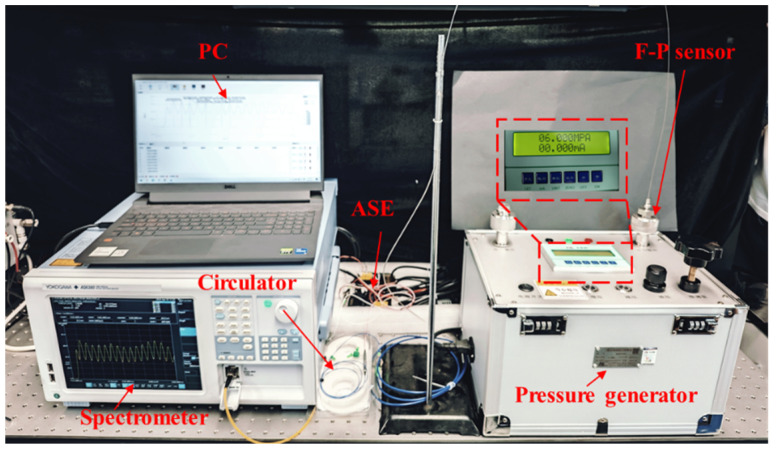
Picture of sensor static performance experimental system.

Using the static performance test system, the pressure measurement range of the pressure sensor can be obtained by comparing data from the sensor sample with data from the standard pressure gauge. The static performance test system for sensors consists of a broadband light source (BEGOLD-ASE-C, Xiamen Beogold Technology Co., Ltd., Xiamen, China), a spectrometer (AQ6380, Yokogawa Electric Corporation, Nakamachi, Musashino City, Tokyo, Japan), a pressure generator (AILEIKE-ALKD1012A, Xiamen Hualian Semiconductor Technology Co., Ltd., Xiamen, China), and an optical circulator. The field system is built as shown in Figure 19.

**Figure 20:**
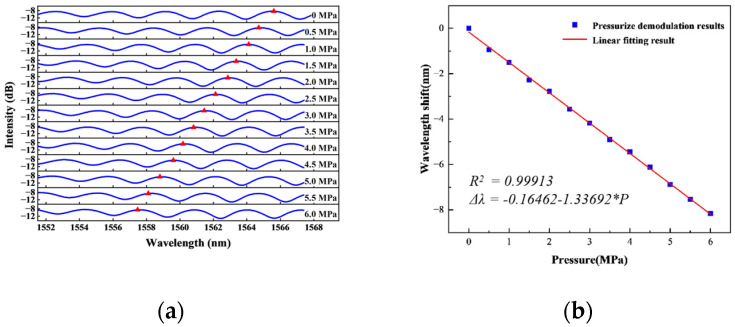

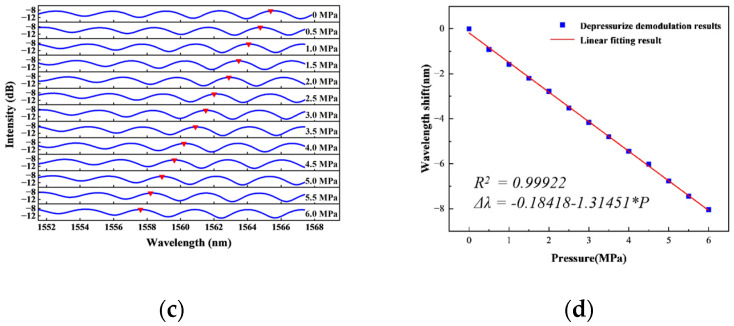
Static experimental results of the sensor: (**a**) Changing trend of spectrum as the pressure increases; (**b**) Wavelength shift and fitting result; (**c**) Changing trend of spectrum as the pressure decreases; (**d**) Wavelength shift and fitting result.

The pressure measurement accuracy of the pressure generator can reach 0.05% Full Scale (F.S.) and the resolution is 1 Pa. Before the start of the static pressure experiment, the sensor is installed to the calibration interface of the pressure sensor through the thread and the rubber sealing ring, and the pressure is loaded to 6 MPa to stand still. During the experiment, the control pressure generator gradually increases the pressure on the sensor with a step of 0.5 MPa. The pressure is increased from 0 MPa to 6 MPa, and then gradually decreased from 6 MPa to 0 MPa, with the same amount of steps. Hold for 60 s at each pressure point to ensure that the sensor output is stabilized, record the sensor output spectrum and demodulate it. The trend of spectral shift in the static experiments of boosting and depressurizing, and the relationship between the wavelength shift obtained by the peak tracking method and the pressure change are shown in Figure 20.

From the experimental results, it can be observed that the interference spectrum of the sensor is shifted to the left as the loading pressure gradually increases. When the pressure change is 6 MPa, the spectrum is shifted to the left by 8.16 nm relative to the standard atmospheric pressure state, and the sensitivity is 1.33692 nm/MPa. On the contrary, when the pressure decreases, the spectrum shifts to the right. The overall shift is 8.04 nm, and the sensitivity is 1.31451 nm/MPa. Based on the linear fitting results, it can be found that the sensor exhibits good linearity in the lift pressure experiments, and the goodness of fit R^2^ is greater than 0.99913 in all cases.

**Figure 21:**
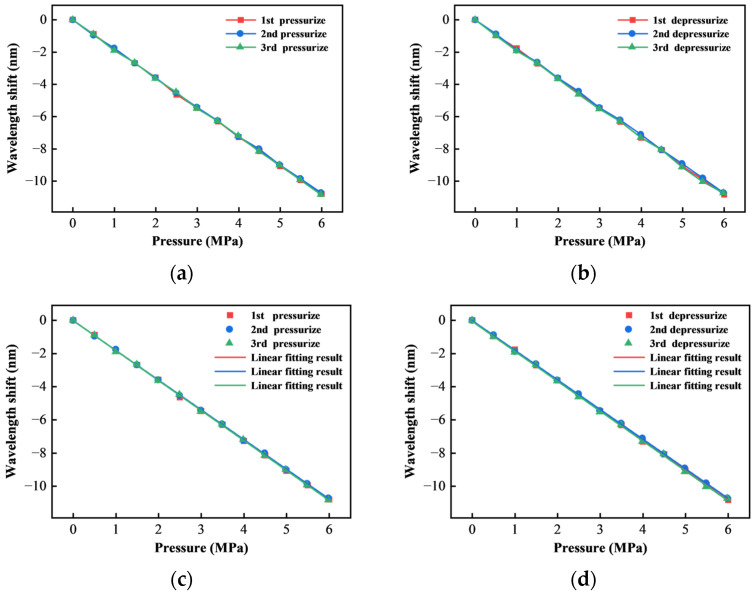
Sensor three times static pressure repeatability curve and linear fitting results: (**a**) Pressurize repeatability curve; (**b**) Depressurize repeatability curve; (**c**) Linear fitting results of pressurize curve; (**d**) Linear fitting results of depressurize curve.

Repeating the above experiments and data analysis methods, the curves of the three times repeatability static pressure measurement experimental results of the sensor and the corresponding linear fitting relationship are obtained as shown in Figure 21.

The statistics of the linear fitting results of the three lift pressure experiments of the sensor are shown in Table 1. The slopes in the table respond to the corresponding magnitude of the shift in the spectra of the sensor per unit of pressure. And R^2^ and residual sum of squares (RSS) are used to characterize the goodness of fit of the data.

According to Table 1, in the three repeated experiments, the slope of the linear fitting curve of the sensor is about 1.80. The average sensitivity is 1.801 nm/MPa. The R^2^ of all fitting results is better than 0.99956.

By analyzing the experimental results, it can be seen that the sensor has good repeatability, and there is no obvious drift in the peak-valley position. It shows that the hysteresis phenomenon caused by multiple rounds of pressure experiment cycle can be ignored.

According to the wavelength–pressure calibration coefficient of the pressure sensor, the pressure value is obtained through calculation and compared with the indication of the standard pressure gauge. The absolute value of the maximum full-scale error is 0.7%, as shown in the Table 2.

### 4.2. Dynamic Experiment

The main purpose of the dynamic performance experiment is to evaluate the frequency response characteristics of the sensor. The core equipment of the sensor dynamic performance test experimental system is the shock tube. When the shock tube is working, the external gas source continues to inflate into the high pressure chamber. When the pressure inside the high-pressure chamber reaches the critical pressure of the bursting diaphragm, the diaphragm breaks, resulting in instantaneous expansion of the gas in the high-pressure zone, forming a high-speed propagation of the shock wave to the low-pressure zone [36].

**Figure 22:**
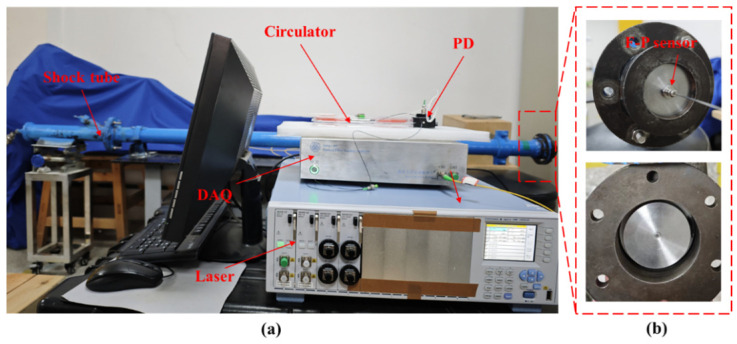
Picture of sensor dynamic performance experimental system: (**a**) Shock tube and acquisition system; (**b**) Sensor installation location.

Since the excitation time is very fast, on the order of milliseconds or microseconds, the single wavelength light intensity method is used for data acquisition and demodulation [37]. The signal acquisition system based on the single-wavelength intensity method is composed of a laser source (AQ2200, Yokogawa Electric Corporation, Nakamachi, Musashino City, Tokyo, Japan), circulator, photodetector (Thorlabs-PDA20CS2, Thorlabs, Inc., Newton, NJ, USA), high-speed signal acquisition card (AlazarTech-Ats935, Alazar Technologies, Inc., Pointe-Claire, QC, Canada) and computer. The data sampling rate of the system can be realized by configuring the signal acquisition card in the upper computer program. The sampling rate in the experiment is 5 MHz. The physical diagram of the dynamic performance experimental system is shown in Figure 22. The static operating point of the single wavelength method is selected by using the results of the static experiment. At the same time, the appropriate critical pressure is generated by controlling the thickness of the blasting diaphragm, so as to ensure that the dynamic response process of the sensor is in the linear working area.

**Figure 23:**
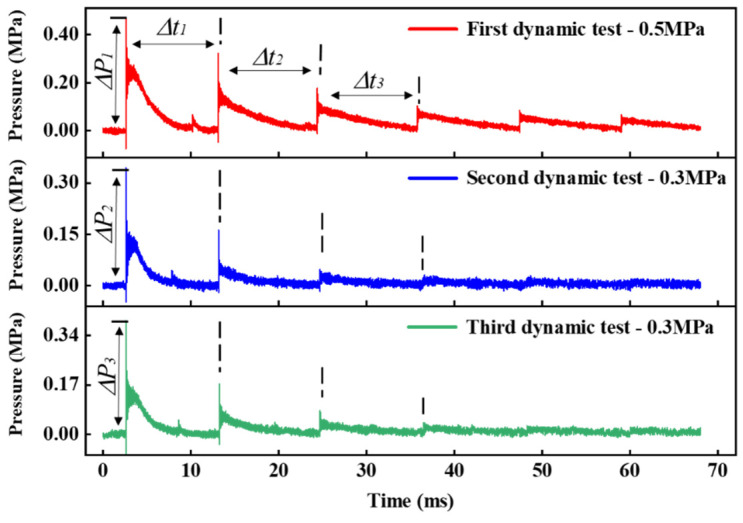
HTCC diaphragm F–P pressure sensor dynamic performance measurement results.

Three dynamic pressure experiments were carried out on the sensor. The excitation pressures controlling the output of the shock tube were 0.5 MPa, 0.3 MPa and 0.3 MPa, respectively. The dynamic response curve of the sensor collected by the system is shown in Figure 23.

**Figure 24:**
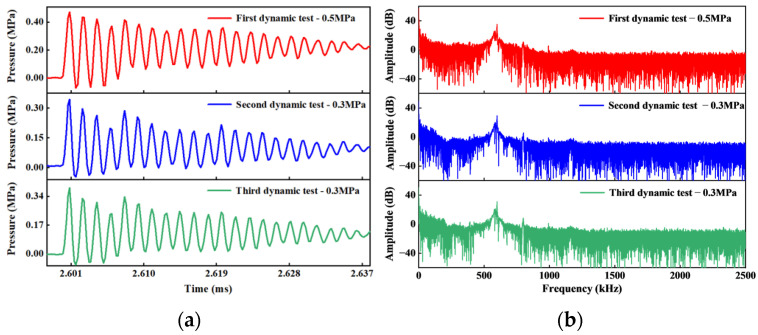
HTCC diaphragm F–P pressure sensor resonant frequency test results: (**a**) Diaphragm resonance response curve; (**b**) FFT spectrum analysis results of resonance response curve.

When the shock wave acts on the sensor, the sensor responds quickly and reaches the set peak pressure. Subsequently, the shock wave is reflected by the end face of the sensor, the pressure effect disappears, the diaphragm rebounds, and the pressure measured by the sensor gradually decays to the initial state. The shock wave will be repeatedly reflected and propagated in the tube, and the sensor can measure multiple shock wave action periods. The burst pressure of the diaphragm is an empirical value, and the shape of each burst film is not consistent, resulting in a difference between the measured peak and the set value. Owing to the periodic dependence of the interference signal on cavity length, Fabry–Perot interferometric sensors are susceptible to fringe skipping during dynamic measurements when spectral shifts exceed the free spectral range. However, in this study, the variation in cavity length remains continuous and monotonic under the applied pressure change rates and dynamic loading frequencies, effectively preventing phase aliasing. In addition, the sensor’s structural response bandwidth exceeds the dominant excitation frequency, ensuring an accurate representation of the system’s true dynamic behavior. The test results of the resonant frequency of the sensor are shown in Figure 24.

Figure 24a is the amplification diagram of the sensor response curve at the peak of the first action period. It can be clearly seen that the response curve is composed of regular oscillating waves. These oscillating waves are the resonant signals generated by the sensor under the action of the shock wave. The results of the spectrum analysis can be obtained as shown in Figure 24b. It can be observed that the spectrum of the sensor response curve has an obvious peak near 598 kHz, which is the resonant frequency of the sensor. Therefore, the HTCC Fabry–Perot pressure sensor has good dynamic performance.

### 4.3. High-Temperature Experiment

**Figure 25:**
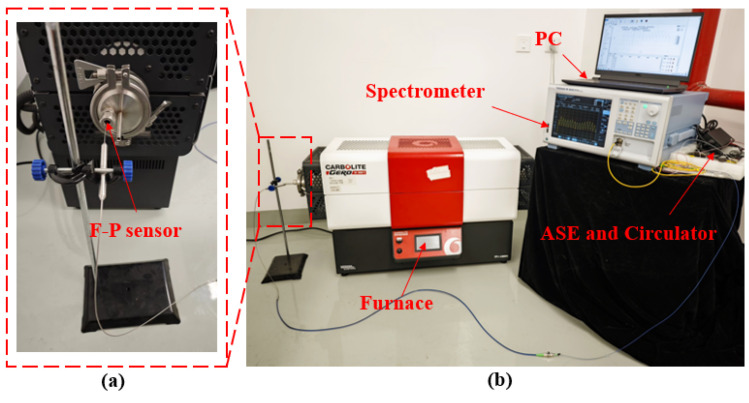
Picture of sensor high-temperature performance experimental system: (**a**) Sensor installation location; (**b**) High-temperature experimental system.

In order to verify the performance of the sensor in a high-temperature environment, we built a high-temperature performance verification experimental system. The system consists of a broadband light source (BEGOLD-ASE-C, Xiamen Beogold Technology Co., Ltd., Xiamen, China), a spectrometer (AQ6380, Yokogawa Electric Corporation, Nakamachi, Musashino City, Tokyo, Japan) and a tubular high-temperature furnace (TF1, Carbolite Gero Ltd., Hope Valle, UK), as shown in Figure 25.

**Figure 26:**
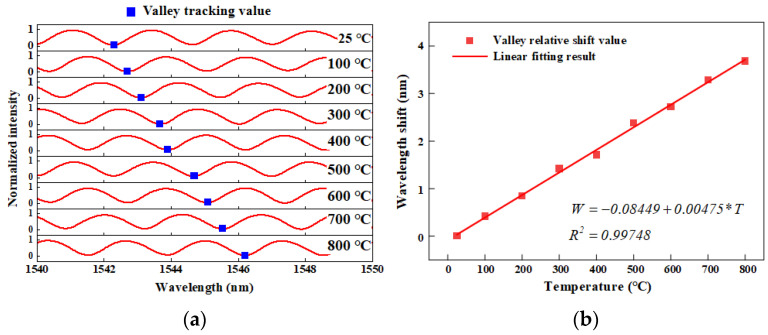
High-temperature experimental results of F–P pressure sensor: (**a**) Sensor interference spectrum changing trend with temperature; (**b**) Sensor marker peak wavelength shift value with temperature and linear fitting results.

The method of the high-temperature experiment is as follows: The HTCC optical fiber Fabry–Perot pressure sensor is installed in a tubular high-temperature furnace, and the heating program is set to control the high-temperature furnace to gradually increase from room temperature to 800 °C at a rate of 5 °C per minute. Insulation was carried out for 60 min at each temperature gradient of 100 °C to ensure stable temperature and sensor output in the furnace. Subsequently, the interference spectrum at each temperature gradient was collected by a spectrometer and the data were processed. The experimental results are shown in Figure 26.

From Figure 26a, it can be seen that when the ambient temperature gradually increases, the interference spectrum of the sensor shifts to the right, which is opposite to the trend when the pressure is applied. This is because when the temperature rises, the structure inside the sensor will produce thermal expansion, resulting in an increase in the length of the Fabry–Perot cavity. In the case of a temperature change of about 775 °C (room temperature 25 °C to 800 °C), the spectrum is shifted to the right by 3.679 nm compared with the normal temperature, and the temperature sensitivity is 0.00475 nm/°C. The linear goodness of fit R^2^ is greater than 0.99748, indicating that the cavity length and structural changes caused by thermal expansion of the sensor at a high temperature are basically linear, which is helpful for temperature compensation and calibration of the sensor.

**Figure 27:**
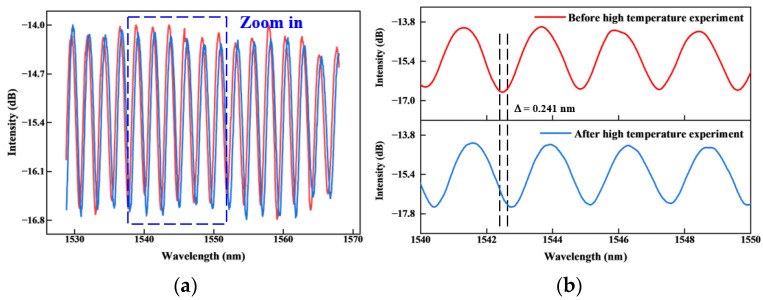
Spectrum comparison before and after the high-temperature experiment: (**a**) Spectrum comparison; (**b**) Spectral enlargement (1540–1550 nm).

Figure 27 shows the comparison of the sensor’s output spectra at room temperature (25 °C) before and after exposure to a high-temperature environment (800 °C) for over 80 h. As shown in Figure 27a, the output spectra before and after prolonged high-temperature testing exhibit good consistency in waveform. The spectrum is basically reunited without obvious change and attenuation. This indicates that the sensor based on a ceramic-based high-temperature sintering process integration has good stability. However, as seen in the magnified view in Figure 27b, a slight spectral shift of approximately 0.241 nm is observed. It shows that some components have irreversible thermal strain after working in a long-term high-temperature environment. The sensor is not fully restored to the initial state. Further investigation is needed to decouple and analyze this effect.

During the thermal cycling test, we observed an overall spectral shift of approximately 0.241 nm. It can be seen that hysteresis is inevitable after repeated experiments. Because the offset is small and tends to be stable after cycling, it shows that the sensor still has good stability and repeatability. In the follow-up research process, we can try to take measures, such as writing gratings in the optical fiber, to achieve temperature compensation.

## 5. Conclusions

In this paper, we propose a scheme of high-temperature pulsating pressure sensor based on HTCC and high-temperature sintering process of an all-ceramic fiber Fabry–Perot cavity. We designed and fabricated the pressure-sensing diaphragm of the concave structure sensor, and proposed an integrated packaging method based on the high-temperature sintering process. In the large pressure range of 0–6 MPa, the static pressure sensitivity of the sensor is 1.30 nm/MPa, and the linear goodness of fit is 0.99913. The resonant frequency of the sensor reaches 598.5 kHz. The survival time at high temperature of 800 °C is more than 80 h. The sensitivity to temperature is 0.00475 nm/°C. In this work, an optical fiber Fabry–Perot pressure sensor with high-temperature resistance and high-frequency response is designed and manufactured, which meets the test requirements of actual project engineering. It provides an effective scheme for the detection of high-speed dynamic changes in the internal airflow of aero-engines.

## Figures and Tables

**Table 1:** Linear fitting results of pressure experiments for sensor.

Pressure Experiments	First Pressurization	First Depressurization	Second Pressurization	Second Depressurization	Third Pressurization	Third Depressurization
Slope	1.807	1.792	1.809	1.806	1.788	1.804
R^2^	0.99984	0.99976	0.99982	0.99987	0.99982	0.99956
RSS	0.02312	0.01828	0.02743	0.01979	0.02680	0.06500

**Table 2:** The static test error of Fabry–Perot pressure sensor.

Standard Pressure (MPa)	Measure Pressure (MPa)	Test Error (F.S.)	Standard Pressure (MPa)	Measure Pressure (MPa)	Test Error (F.S.)
0	0.019	0.3%	3.5	3.514	0.2%
0.5	0.480	−0.3%	4	3.996	−0.1%
1	0.962	−0.6%	4.5	4.481	−0.3%
1.5	1.473	−0.4%	5	4.977	−0.4%
2	1.994	−0.1%	5.5	5.532	0.5%
2.5	2.522	0.4%	6	5.959	−0.7%
3	3.037	0.6%	-	-	-

## Data Availability

The original contributions presented in this study are included in the article. Further inquiries can be directed to the corresponding author.
